# Assessing urban park equity in Chaoyang District, Beijing using online review data

**DOI:** 10.1038/s41598-024-51239-9

**Published:** 2024-01-12

**Authors:** Ning Zou, Xiayuan Mi, Yang Xiao, Yunyuan Li, Nan Hu

**Affiliations:** School of Landscape Architecture, Beijing Forestry University, Beijing, 100083 China

**Keywords:** Urban ecology, Sustainability, Socioeconomic scenarios

## Abstract

Urban parks are essential components of urban ecosystems, providing vital ecological resources for city residents. However, the rapid expansion of high-density urban areas has led to an unequal distribution of park resources, raising growing concerns about spatial equity. To address these challenges, we employed an improved Gaussian two-step floating catchment area (2SFCA) method, considering park quality variations and integrating sentiment scores from park reviews to calculate a comprehensive park accessibility index, accounting for both supply and demand dynamics among park users. The results demonstrate the significance of park management, as users prioritise convenience and cleanliness of public facilities. Recreational quality significantly influences park distribution equity, with areas near Beijing’s initial greenbelt zone showing improved accessibility (IA). Nonetheless, our analysis exposes disparities in urban park resource allocation within the Chaoyang District, indicating relative inequity. Spatial supply and demand mismatches, especially in the northwest and southeast, are evident. To enhance park layout equity, we recommend strategies like identifying and repurposing underused spaces, establishing pocket parks and micro-green areas, and improving recreational facilities. It is crucial to address the needs of vulnerable groups such as older residents and children. These insights stress the importance of ensuring fair urban park access to enhance the well-being of all city residents.

## Introduction

Urban parks serve as integral elements of urban green infrastructure^1,2^, playing a crucial role in sustaining urban ecosystems^3,4^. Not only do they provide valuable ecological services, they also offer urban residents greater opportunities to connect with nature,^5–8^, alleviating psychological stress, promoting increased social interaction, and supporting fitness and leisure activities^9^, further ensuring the physical and mental well-being of urban communities^10–13^. However, as China’s urbanisation process accelerates, the population of Beijing has reached a staggering 21.843 million people. As a representative of the core city cluster in terms of economic development, Beijing faces a range of challenges relating to high-density development, such as uncontrolled land expansion and excessive population concentration^14^. In densely populated urban environments, the condensed spatial configuration, combined with high population density and limited land resources^15^, has led to a reduction in the quantity and a deterioration in the quality and functionality of urban parks^16–18^. These factors have had a detrimental impact on the provision of urban green spaces, with many residents unable to enjoy equal access to green resources. To alleviate this pressure regarding resource distribution disparity in high-density urban parks, it is necessary to evaluate the supply capacity of urban park resources, considering both spatial distribution and park quality, while adhering to stock development planning policy^19^. Merely evaluating urban parks supply capacity through an analysis of spatial distribution patterns is inadequate to address the contemporary needs of densely populated cities^20^. Stock development primarily focuses on enhancing the quality of limited available space. Therefore, it is crucial to integrate park quality evaluations to comprehensively assess the supply–demand relationship between urban parks and users.

Recent studies concerning the equity of urban parks have increasingly considered the impact of inherent differences in park quality on resource distribution equity. For instance, some studies have incorporated quantitative indicators of the cooling impact of green park areas to adjust the parameters used in accessibility calculations and have combined these indicators with demand factors to evaluate the equity of cooling services provided by parks within a given region^21,22^. Some researchers have focused on improving accessibility analysis methods by incorporating multiple dimensions of park quality evaluation, including availability, attractiveness, and aesthetics when assessing the equity of urban parks^23,24^. Several research endeavors have also investigated the influence of various modes of transportation, as well as the uneven quality of urban green spaces when determining equity considerations^25^.

Traditional analytical methods regarding the spatial equity of urban park distribution typically use the park accessibility index as the supply measure and population size as the demand measure. Assessments are conducted by analysing the ratio between the two^26–28^. Accessibility pertains to the residents’ capability to overcome challenges such as distance and time when reaching their intended destination. It is an effective method for evaluating whether the distribution of urban public resources meets the relevant demand needs^29,30^. Common methods for measuring accessibility include buffer zone analysis^31^, gravity models^32^, minimum distance^33^, network analysis^34^, cost-weighted distance^35^, and the Gaussian two-step floating catchment area (G2SFCA) method^36^. Among these approaches, the G2SFCA method, in particular, comprehensively considers supply and demand and calculates the accessibility of public service facilities using two steps^27,37^. This method can be optimised and expanded by adjusting the distance decay functions, search radius, and other parameters^38,39^, and is widely applied in accessibility evaluations owing to its intuitive expression, simple computation, and strong operability.

Considering the impact of inherent differences in urban park quality on equity, combining equity research with quality analysis can further enhance the reliability of the research methods and results. As visitors are both users and evaluators of parks, analysing visitor evaluations can offer a more holistic insight into the extent of public contentment with park recreational quality^40^, thus playing a crucial role in assessing inherent differences in park quality^41,42^. Compared to traditional questionnaire surveys, evaluation data from social media are more easily accessible and offer larger sample sizes. With the development of network technology, combining software programs to analyze big data has become a popular trend and is being applied in various research fields^43,44^. Previous studies have utilised big data from online media platforms to conduct semantic analysis for evaluating park recreational quality^45–47^. However, there is limited research that incorporates the quantified data from semantic analysis into the calculation of park accessibility.

The population density in Chaoyang District, Beijing is approximately 7564 people per square kilometer, with a higher concentration of population on the western side of the urban area. To alleviate the loading pressure in the district and improve the quality of residents’ living spaces, the district’s zoning plan proposes suggestions such as optimising park layouts, enhancing park service efficiency, and emphasising the characteristic development of parks^48^. This study first analyzes 96,220 evaluations of parks in Chaoyang District with 43 blocks, obtaining sentiment scores for 83 parks. These sentiment scores are then incorporated into an accessibility calculation formula, using an improved accessibility (IA) index and approximately 55,797 community population data to provide a more accurate assessment of the social equity of parks in Beijing’s Chaoyang District accurately. By doing so, it will provide valuable insights for other high-density urban areas facing similar situations, offering guidance on optimising park layouts, enhancing service efficiency, and emphasising characteristic park development.

The article consists of an introduction, methodology, result, discussion, and conclusions. The introduction presents the research hotspots, analysis methods, and study framework. The “Methodology” section describes the research subjects and analysis methods. The “Result” section includes street-level maps and data summaries. The “Discussion” section provides suggestions based on the results. The “Conclusions” summarizes the experiment, results, innovation, challenges, and future research possibilities.

## Methodology

### Study area

**Figure 1:**
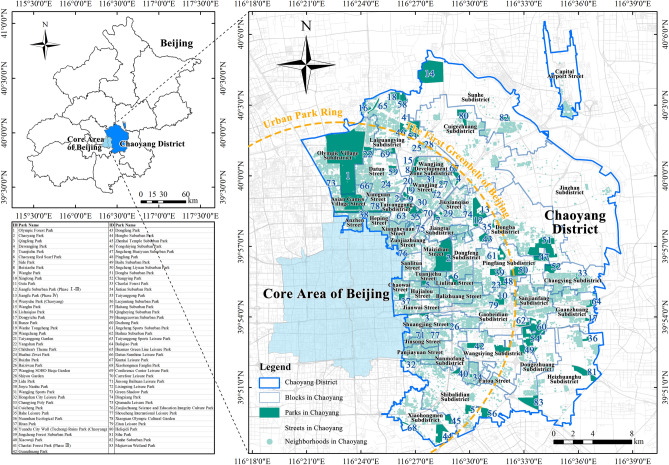
Study area.

The primary emphasis of this investigation was placed on 83 urban parks in the Chaoyang District of Beijing, as listed in the “Beijing Park Directory (First Batch)”^49^. Administrative blocks within the district were selected as the spatial units for comparative analysis. Located in the southern segment of Beijing’s primary urban zone (39°49′–40°5′ N, 116°21′–116°38′ E), Chaoyang District covers an area of 470.8 km^2^. It consists of 43 blocks and has a permanent population of 3.449 million people^50^. As of 2022, its GDP reached 791.12 billion yuan^51^, making it the largest and most densely populated district in Beijing. The per capita park area in the district is currently 18.54 m^2^, and the coverage rate of park coverage within a 500-m service radius is 88.91%^52^. These figures indicate that the management of green spaces in Chaoyang District has achieved a certain degree of success (Fig. 1).

### Research method

**Figure 2:**
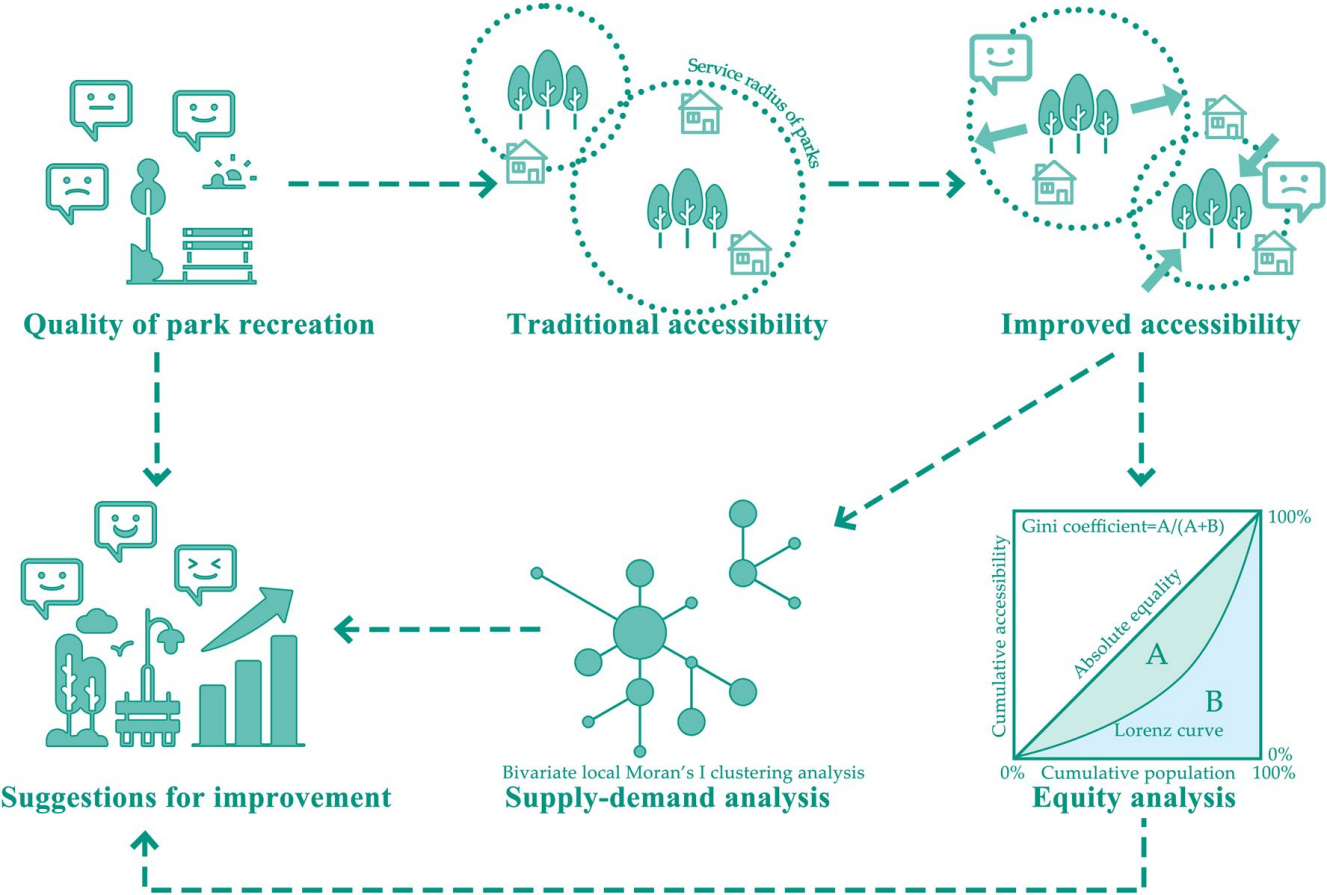
Research method flow.

This study used Chaoyang District in Beijing as the research sample area and utilised the SnowNLP package in Python for the semantic sentiment analysis of online media reviews. The perceived emotional scores of visitors towards the parks were used as the evaluation criteria for park recreational quality. Subsequently, by incorporating park sentiment scores into G2SFCA accessibility calculations, the integrated accessibility index, which considers the quality of park recreation, was used to measure the supply capacity of park resources in Chaoyang District. Furthermore, park distribution equity was analysed by combining different user data at the block level. Finally, by integrating the results for park accessibility, spatial equity, and recreational quality analyses, optimisation, appropriate policy suggestions were provided (Fig. 2).

### Park recreational quality evaluation

User reviews were gathered from China's most popular tourist review websites, including Dianping, Ctrip, and Mafengwo, to obtain detailed feedback on the urban parks in Chaoyang District. The collected data included user IPs, review timestamps, and content. After filtering out irrelevant reviews, such as advertisements and web links, a total of 96,220 textual reviews were obtained.

A sentiment analysis was conducted of the obtained review texts using the SnowNLP library in Python. SnowNLP is a toolset founded on a Bayesian model tailored for the purpose of conducting natural language processing and sentiment analysis in the Chinese language^53^. The formula for the sentiment analysis was as follows:

$$P(c_{i} |w_{1} ,...,w_{n} ) = \frac{{[P(w_{1} ,...,w_{n} |c_{i} ) \times P\left( {c_{i} } \right)]}}{{[P(w_{1} ,...,w_{n} |c_{1} ) \times P\left( {c_{1} } \right) + P(w_{1} ,...,w_{n} |c_{2} ) \times P\left( {c_{2} } \right)]}},i = 1,2$$

where $c_{1}$ and $c_{2}$ represent the positive and negative sentiment of the sample, respectively. $w_{n}$ represents the probability of a specific sentiment feature word $w$ appearing in the test sample, and $P\left( {c_{i} } \right)$ represents the probability of a sentence with the feature $w_{n}$ belonging to sentiment class $c_{i}$.

The programming technique used references from the English natural language processing library TextBlob but did not cite the Natural Language Toolkit (NLTK) library. To improve the accuracy of sentiment analysis in SnowNLP, 4336 comment texts were randomly extracted from the source data and classified into positive and negative sentiments. The classification results were used to train SnowNLP for sentiment analysis. Additionally, stop words were incorporated to avoid unnecessary interference from certain vocabulary items in the analysis results. The segmentation of the evaluation texts was also optimised by combining them with the Jieba word segmenter. The sentiment analysis output values range was between 0 and 1. A value closer to 1 indicated a more positive sentiment in the comment, whereas a value closer to 0 suggested a more negative sentiment^54^.

To explore the key elements of the park that influence visitors' recreational experiences, in this study, texts were categorized as expressing positive emotions if their sentiment scores were greater than or equal to 0.5, while those with scores less than 0.5 were deemed to convey negative emotions. The Jieba Chinese word segmenter was used to analyse the word frequency in both positive and negative sentiment texts. The key elements affecting visitors' emotions were identified through this analysis. These findings serve as a reference point for proposing strategies to enhance the overall experience of park leisure and set the foundation for suggesting optimisation strategies for park equity in Chaoyang District.

### Improved accessibility analysis

The G2SFCA approach employs a Gaussian function as the distance decay model, with low decay rates for short and long distances, and a notable decay rate in the intermediate distance range. This method comprehensively considers the interactions between supply and demand. It sequentially sets the supply and demand points as centres and draws service radii, conducting two rounds of searching to determine the accessibility of public facilities^36^.

In the first step, entrance/exit point $j$ of the park was considered the supply point. A threshold $d_{0}$ was set based on the size and type of the park to establish a search domain. Within this search domain, the community demand points $k$ were identified, and the total population within the search domain was aggregated. The supply–demand ratio $R_{j}$ of the park was calculated as follows:

$$R_{j} = \frac{{S_{j} }}{{\mathop \sum \nolimits_{{k \in \left\{ {d_{kj} \le } \right.\left. {d_{0} } \right\}}} G\left( {d_{kj} ,d_{0} } \right)P_{k} }}$$

$$G\left( {d_{kj} ,d_{0} } \right) = \left\{ {\begin{array}{ll} {\frac{{e^{{ - \left( \frac{1}{2} \right) \times \left( {\frac{{d_{kj} }}{{d_{0} }}} \right)^{2} }} - e^{{ - \left( \frac{1}{2} \right)}} }}{{1 - e^{{ - \left( \frac{1}{2} \right)}} }}, \quad d_{kj} \le d_{0} } \\ 0, \qquad \qquad\qquad \qquad d_{kj} > d_{0} \end{array} } \right.$$

where $d_{kj}$ represents the spatial separation from supply point $j$ to demand point $k$. $d_{0}$ represents the service radius of the park. $S_{j}$ represents the park area. $P_{k}$ represents the population at demand point $k$. $G\left( {d_{kj} ,d_{0} } \right)$ represents the Gaussian distance attenuation function.

In the second step, a search domain was established around each community demand point $k$. The supply demand ratios $R_{j}$ of supply point $j$ within the search domain were summed using a Gaussian decay function. This yielded park accessibility $A_{k}^{F}$ for demand point $k$, where a higher value signified increased accessibility. The formula was as follows:

$$A_{k}^{F} = \mathop \sum \limits_{{j \in \left\{ {d_{kj} \le d_{0} } \right\}}} G\left( {d_{kj} ,d_{0} } \right)R_{j}$$

where $d_{jk}$ represents the distance from demand point $k$ to supply point $j$. $d_{0}$ represents the travel capacity of residents to the urban park. $S_{j}$ represents the park area. $P_{k}$ represents the population at demand point $k$. $G\left( {d_{kj} ,d_{0} } \right)$ represents the Gaussian distance decay function.

To ensure the validity of the experiment, data regarding community locations and household numbers were collected for Chaoyang District. The data were then cross-referenced with the 7th National Census data at the block level to adjust the population count for each community. When delineating the search domains, we took into account the maximum network distance that residents would travel to reach the park, as opposed to a straightforward linear distance. The search domains for the same park entrances/exits were also merged. The residents' travel capacity was based on the 15-min living circle planning recommendations, with walking, cycling, and driving travel distances set at 1.25 km, 3.75 km, and 7.5 km, respectively. The park service radius ${d}_{0}$ was determined based on the results from the relevant literature^20,55–57^ and the “Urban Green Space Classification Standards”. The specific radius was set according to the type and level of park (Table 1). In the traditional approach, $S_{j}$ represents the park area. However, in the improved method, the indicators for evaluating the quality of park recreation are incorporated. $S_{j}$ is a measure of the park's supply capacity, reflecting its recreational quality.

$$S_{{j\left( {RQ} \right)}} = S_{j} \left( {1 + x_{EM} } \right)$$

**Table 1:** Service radius of urban parks in Chaoyang District.

Park type	Park level	Service radius/m^2^
Comprehensive Park	First level	5000
	Second level	3000
Historical Park	First level	5000
Specialised Park	First level	5000
	Second level	3000
	Third level	1000
Community Park	Second level	1000
	Third level	500
Ecological Park	Second level	1000
	Third level	500
Recreational Garden	Third level	500
	Fourth level	300

In the above equation, $x_{EM}$ represents the visitor’s sentiment scores for their overall park experience, whereas $S_{{j\left( {RQ} \right)}}$ is considered an indicator that reflects the park’s supply capacity in terms of recreational quality.

### Equity evaluation

#### Lorenz curve and Gini coefficient

Equity in the distribution of urban park resources across diverse demographic groups in Chaoyang District was evaluated through the utilization of the Lorenz curve and the Gini coefficient. The horizontal axis represented the cumulative percentage of the resident population, while the vertical axis depicted the cumulative percentage of accessibility to urban parks. The ratio of the coordinates on the horizontal and vertical axes equals 1 and represents the absolute equality line, indicating a state of absolute equity in terms of park resource distribution. The proximity of the Lorenz curve to the line of absolute equality indicates the level of fairness in the distribution of park resources. The Gini coefficient, which ranges between 0 and 1, is a statistical indicator of equity based on the Lorenz curve. A higher value indicates a greater degree of inequality and curvature in the Lorenz curve (Table 2)^58–60^. The formula for the Gini coefficient is:

$$Gini = 1 - \mathop \sum \limits_{k = 1}^{n} \left( {P_{k} - P_{k - 1} } \right)\left( {R_{k} + R_{k - 1} } \right)$$

where $P_{k}$ represents the cumulative percentage of demand (total population, older population, and child population) for each block, $R_{k}$ represents the cumulative percentage of park accessibility for each block, and $n$ represents the number of blocks.

**Table 2:** Gini coefficient fairness indicators.

Range of Gini coefficient	Distribution of park resources	Equity status
0 ≤ Gini < 0.200	Highly equitable	Extremely equity
0.200 ≤ Gini < 0.300	Relatively equitable	Fairly equity
0.300 ≤ Gini < 0.400	Reasonably balanced	Relatively equity
0.400 ≤ Gini < 0.600	Significant disparities	Fairly inequity
0.600 ≤ Gini < 1.000	Vast disparities	Extremely inequity

#### Bivariate local Moran’s I

Performing a Bivariate local Moran’s I analysis on park accessibility and population density at the block level in Chaoyang District using GeoDa software^61^, the formula is as follows:

$$I_{i} = Z_{xi} \mathop \sum \limits_{{j = 1,{ }j \ne i}}^{n} W_{ij} Z_{yj}$$

where $I_{i}$ represents the local Moran's I (LISA) for block $i$, $W_{ij}$ represents the spatial weight value, $Z_{xi}$ and $Z_{yj}$ represents the standardised values of accessibility and population density in a particular block.

By assessing the level of matching between supply and demand, the assessment outcomes reveal four clustering types for park resource supply capacity: H–H (high–high), H–L (high–low), L–H (low–high), and L–L (low–low) (Table 3).

**Table 3:** Supply–demand matching relationship.

Autocorrelation level clustering type	Variable description			
	Park accessibility	Population density	Matching relationship	Park supply capacity
H–H	High supply	High demand	Spatial positive matching	Supply and demand matching
H–L	High supply	Low demand	Spatial positive mismatch	Oversupply
L–H	Low supply	High demand	Spatial negative mismatch	Insufficient supply
L–L	Low supply	Low demand	Spatial negative matching	Supply and demand matching

## Result

### Evaluation of park recreational quality

#### Sentiment score for review texts

**Figure 3:**
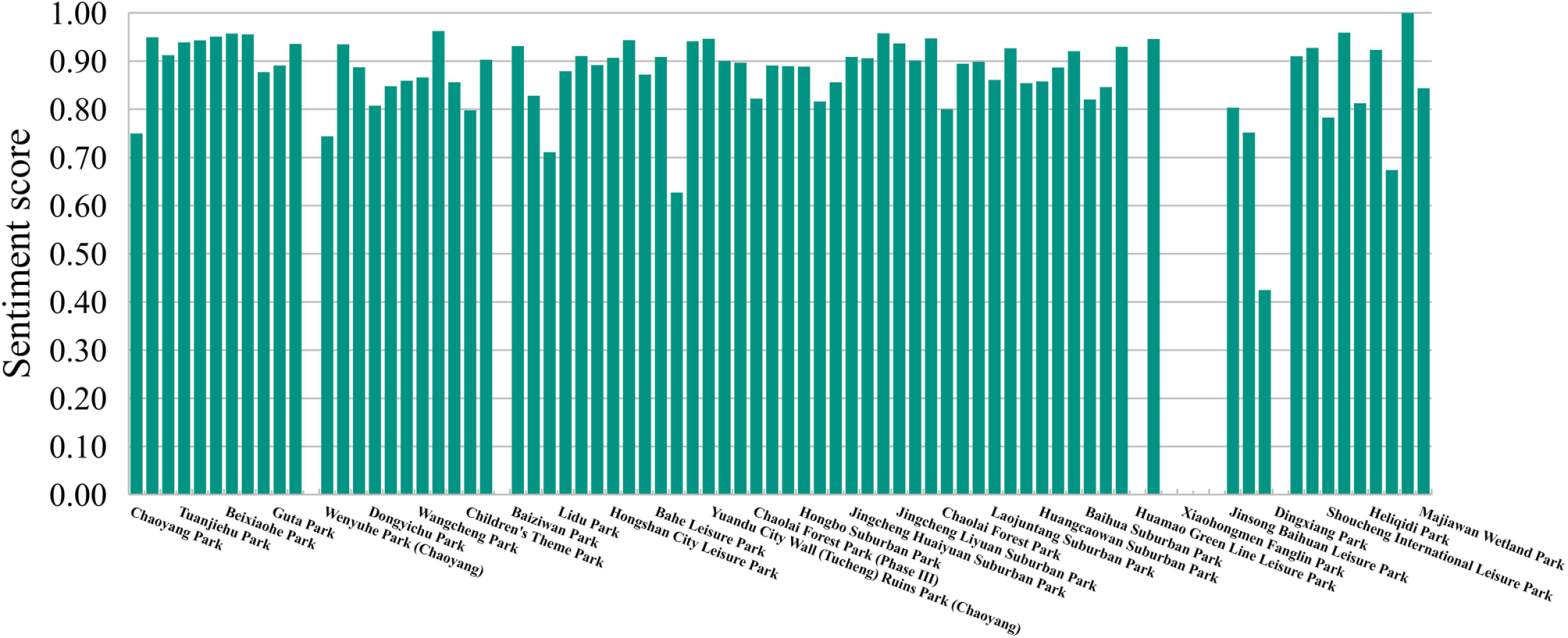
Sentiment score chart of visitors in urban parks of Chaoyang District.

The sentiment scores of the evaluation texts ranged from 0 to 1 (Table 4). By averaging these scores, the sentiment score $x_{EM}$ was obtained for each park (Fig. 3). Among the 75 parks for which evaluation texts were collected, the sentiment scores ranged from 0.992,127,375 to 0.417,140,671. Sunhe Suburban Park received the highest score, whereas Green Shadow Park received the lowest.

**Table 4:** Examples of sentiment analysis in evaluation texts.

Evaluation text	Sentiment score
“朝阳公园”应该是北京四环以内最大的城市公园, 住在附近的人们, 很是幸福啊!园内有丰富的林木和绿地, 可以户外野餐, 放放风筝, 娱乐嬉戏, 好是惬意的!(“Chaoyang Park” is probably the largest urban park within the Fourth Ring Road in Beijing. People who live nearby are truly fortunate! The park is abundant with trees and greenery, providing opportunities for outdoor picnics, kite flying, and recreational activities)	0.999,998,590
12月11日去仰山公园, 发现厕所不能正常使用, 全部是用铁丝把门拧死的, 唯一一个开门的是被人破门的, 里面的水龙头都是坏的。门口的停车位也很少, 只开了大门外面那一片。(During my visit to Yangshan Park on December 11th, I found the toilets to be unusable. All the doors were secured with twisted wire, except for one that had been forcefully broken. The taps inside were also damaged. Moreover, there were limited parking spaces near the entrance, with only the area outside the main gate available)	0.000,000,005

To further investigate the factors influencing visitor perceptions and evaluations, a correlation analysis was conducted between the sentiment scores and other characteristic park attributes (Table 5). The results indicated a significant correlation between the number of park reviews and the park type, level, and size, suggesting that larger-scale and higher-level parks attract more visitors. However, there was no correlation between the number of park reviews and $x_{EM}$, indicating that the popularity of a park does not entirely reflect visitors’ recreational experience or the quality of a park's recreational activities. There was also no correlation between $x_{EM}$ and park size, suggesting that park size was not significantly associated with visitors’ recreational experiences. Moreover, a significant correlation was found between $x_{EM}$ and park type, indicating that comprehensive parks generally received higher sentiment scores in visitor evaluations, whereas recreational gardens received lower scores. Furthermore, $x_{EM}$ was significantly negatively correlated with park level, indicating that lower-level parks received lower sentiment scores in the evaluation texts.

**Table 5:** Correlation between sentiment scores and other park attributes.

		Park type	Park level	Park size	Number of reviews	$x_{EM}$
$x_{EM}$	Pearson correlation	− 0.221*	− 0.281*	0.089	0.122	
	Sig. (two-tailed)	0.045	0.01	0.426	0.272	
	Number of cases	83	83	83	83	
Number of reviews	Pearson correlation	− 0.357**	− 0.418**	0.798**		0.122
	Sig. (two-tailed)	0.001	0	0		0.272
	Number of cases	83	83	83		83

Note: * indicates significance at the 0.05 level (two-tailed), ** indicates significance at the 0.01 level (two-tailed), indicating significant correlation.

#### Frequent words in the comments text

Word frequency analysis was conducted on the review text and categorised into positive and negative sentiments. The analysis sorted the vocabulary based on the frequency of occurrence, and repetitive and irrelevant words were eliminated multiple times to obtain the 100 most frequently occurring words in each category. The results (Table 6) indicate that positive reviews consisted primarily of adjectives and verbs. Frequently used adjectives included cost considerations (免费free), visitor experience (舒服comfortable), and park characteristics (干净clean, 安静quiet). Frequently used verbs addressed leisure activities (拍照photography, 跑步run, 散步walk, 健身exercise, 野餐picnic) and pre-made appointments (预约pre-book). Nouns mainly referred to the natural environment (环境environment, 向日葵sunflower, 树木tree, 草坪lawn, 湿地wetland), transportation facilities (停车场parking lot, 交通transportation), artificial landscapes (广场square, 建筑building, 喷泉fountain), and historical cultural elements (heritage site).

**Table 6:** Partial high-frequency vocabulary.

Frequent vocabulary in positive sentiment reviews	免费(free), 拍照(photography), 跑步(running), 环境(environment), 设施(facilities), 停车场(parking lot), 门票(tickets), 交通(transportation), 疫情(epidemic), 面积(area), 散步(walking), 溜达(strolling), 健身(fitness), 门口(entrance), 秋天(autumn), 小朋友(children), 休闲(leisure), 野餐(picnic), 开放(open), 划船(boating), 锻炼(exercise), 风景(scenery), 银杏(ginkgo), 向日葵(sunflower), 广场(square), 游乐(amusement), 运动(sports)……
Frequent vocabulary in negative sentiment reviews	孩子(children), 停车场(parking lot), 门票(tickets), 停车(parking), 庙会(temple fair), 设施(facilities), 门口(entrance), 免费(free), 自行车(bicycles), 游乐(amusement), 不让(not allowed), 收费(fees), 疫情(epidemic), 帐篷(tent), 划船(boating), 排队(queuing), 环境(environment), 电瓶车(electric scooters), 游乐场(playground), 搭帐篷(setting up tents), 沙滩(beach), 野餐(picnic), 保安(security), 进门(entering), 向日葵(sunflower)……

In contrast, negative comments were primarily defined by the use of nouns, including references to personnel (孩子children, 保安security guard, 工作人员staff, 游客visitor), ticketing (门票entrance ticket, 套票package ticket, 押金deposit, 停车费parking fee), transportation facilities (停车场parking lot, 自行车bicycle, 电瓶车electric scooter, 公交public transportation), cultural events (庙会temple fair), entertainment facilities (游乐场playground, 冰场skating rink), and service facilities (厕所restroom). The adjectives used in negative comments primarily revolved around cost considerations (免费free, 收费charged, 便宜inexpensive) and park characteristics (太大too large, 人多crowded, 没人empty). The verbs used primarily addressed leisure and recreational activities (划船boating, 搭帐篷set up a tent, 拍照photograph), and the subject of making reservations for specific activities was also mentioned (排队queue up, 预约pre-book). The terms “pandemic”(疫情) and “internet celebrity”(网红) were also frequently referenced.

### Improved accessibility

#### Improved accessibility of the community

**Figure 4:**
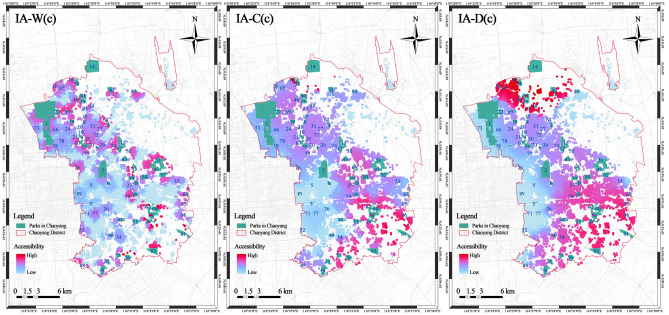
Graded map of improved accessibility of the community.

The level of park accessibility represents how easily they can be reached from community units. A higher accessibility index indicates that urban parks are more easily accessible to a community. By adopting the improved G2SFCA method and analysing the different travel patterns of community residents, the accessibility index of 55,797 community locations in Chaoyang District was calculated (Fig. 4). The results showed that the improved community walking accessibility (IA-W(c)) index range is from 0 to 8,546.390, and there were 21,392 communities with an IA-W(c) index of 0, and that the community with the highest index was located in the Heizhuanghu Subdistrict. Communities with high park accessibility exhibited a chequerboard-like spatial pattern, mainly located around park areas such as the Conference Center Leisure Park, Dawangjing Park, Jiangfu Suburban Park, Dongba Suburban Park, Jintian Suburban Park, Majiawan Wetland Park, and Xiaohongmen Fanglin Park, indicating higher accessibility indices. The reason could be that, owing to travel mode restrictions, residents can only reach parks near their residential areas within a 15-min travel time. Highly accessible communities have a lower population, but greater park supply in their vicinity, which produces this result.

The improved community cycling accessibility (IA-C(c)) index range is from 0 to 42,540.369, and there were 603 communities with an IA-C(c) index of 0, which was significantly lower than that of IA-W(c), indicating a reduced number of communities that could not access any parks. The community with the highest accessibility index was located in the Laiguangying Subdistrict. Communities with high park accessibility exhibited belt-like spatial patterns, with communities in the northwest and southeast exhibiting higher accessibility indices. This could be because residents can reach more parks within the same travel time when using cycling as the travel mode, leading to a more pronounced regional distribution pattern of accessibility indices.

The improved community driving accessibility (IA-D(c)) index range is from 0 to 61,890.425, and there were 51 communities with an IA-D(c) index of 0, and the community with the highest index was located in the Cuigezhuang Subdistrict. High-accessibility areas accounted for a larger proportion, exhibiting a more coherent belt-like spatial pattern, with lower accessibility in the southwest and northeast and higher accessibility in the northwest and southeast. This could be because driving allows access to the majority of the parks in Chaoyang District, resulting in a greater increase in accessibility. This leads to a more pronounced belt-like spatial distribution pattern of highly accessible communities, with sparser residential locations in the northwest and southeast. The parks in these areas are larger and have higher sentiment scores, contributing to higher accessibility indices.

#### Improved accessibility of the block

**Figure 5:**
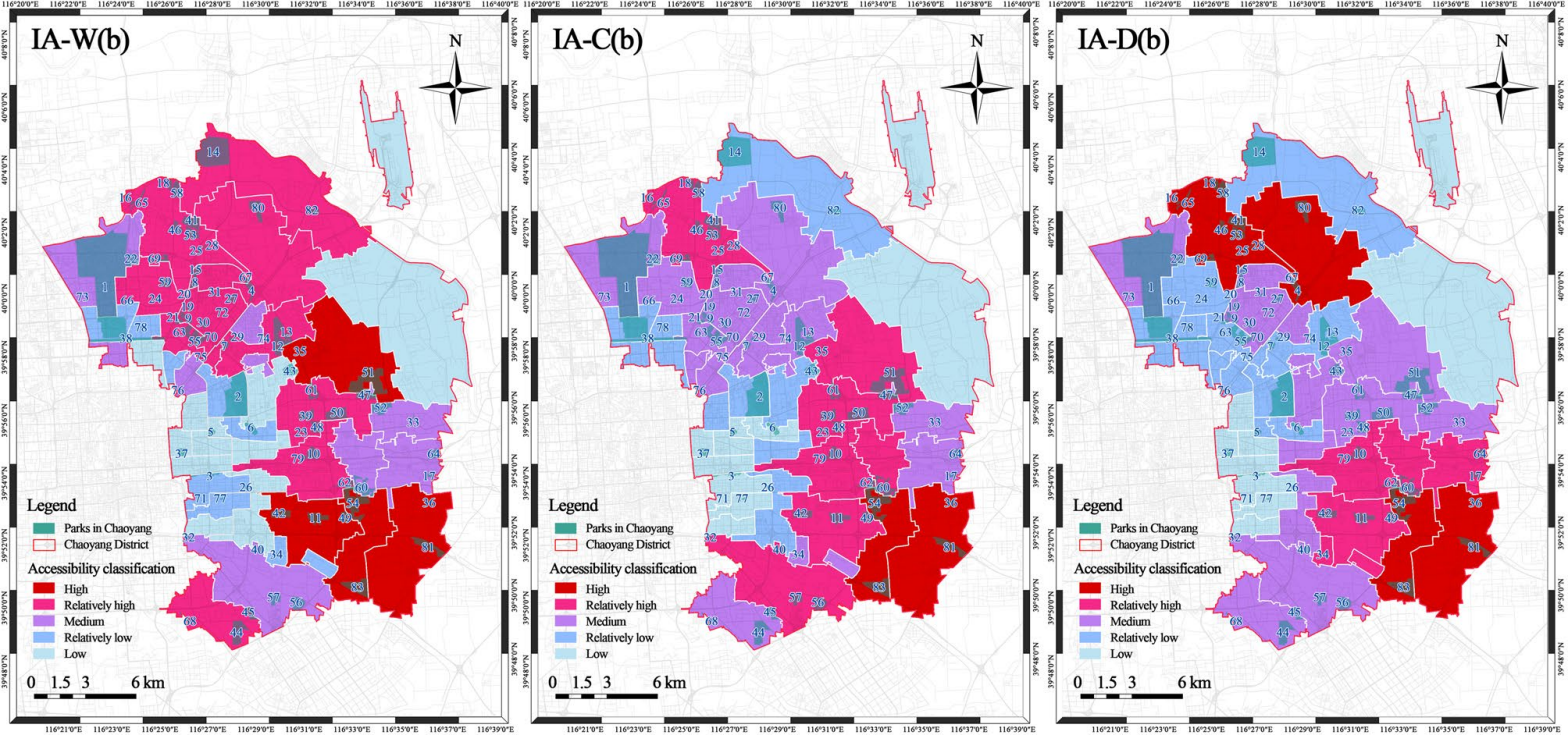
Graded map of improved accessibility of the block.

To facilitate comparative analysis, the average park accessibility values for each street were calculated, and a geometric interval method was used to classify them into five categories: high, relatively high, medium, relatively low, and low (Fig. 5). The results indicated that 4 blocks were classified as high in the improved block walking accessibility (IA-W(b)), including the Dougezhuang Subdistrict (187.786) and Wangsiying Subdistrict (139.120). 11 blocks were classified as relatively high, including the Laiguangying Subdistrict (65.265) and Gaobeidian Subdistrict (52.325). 8 blocks fell into the medium category, including the Changying Subdistrict (27.590) and Guanzhuang Subdistrict (26.971). 8 blocks were classified as relatively low, including Jinsong Street (9.571) and Xiaoguan Street (8.082). 12 blocks fell under the low category, with the lowest IA-W(b) observed in Capital Airport Street (0.000). The results of the analysis suggest that in areas such as the Dougezhuang and Wangsiying Subdistricts, although the population is relatively small, there are a greater number of parks distributed there. Due to travel mode restrictions, residents tend to visit parks closer to their homes, resulting in a relatively higher distribution of parks per capita and higher walking accessibility. Conversely, Capital Airport Street has a smaller population, but is furthest from the urban park locations, with fewer access routes, leading to the lowest park accessibility in that area.

In terms of the improved block cycling accessibility (IA-C(b)) classification, there are 2 blocks classified as high: Heizhuanghu Subdistrict (4,266.356) and Dougezhuang Subdistrict (3,548.887). 7 blocks were classified as relatively high, 14 as medium, 10 as relatively low, and 10 as low. The lowest IA-C(b) value was observed in Capital Airport Street (0.000), followed by Jinzhan Subdistrict (11.620) and Hujialou Street (14.209). Compared with walking, fewer blocks were categorised as having low accessibility using cycling as the mode of transportation. The reason for this could be that blocks with a higher population, such as the Shibalidian and Sanjianfang Subdistricts, have a lower per capita distribution of park resources for walking. However, these blocks also have a higher number of surrounding parks, meaning the 15-min cycling distance allows access to a greater number of parks than walking.

In terms of the improved block driving accessibility (IA-D(b)) classification, there are 4 blocks classified as high, including the Laiguangying Subdistrict (24,156.163) and Cuigezhuang Subdistrict (14,972.491). 5 blocks were classified as relatively high, 12 as medium, 13 as relatively low, and 9 as low. The lowest IA-D(b) value was observed in Capital Airport Street (41.641). The reason for this could be that areas with higher populations, such as the Laiguangying and Cuigezhuang Subdistricts, have relatively fewer parks. As a result, residents in these areas can reach most of the urban parks in Chaoyang District within a 15-min drive, thus increasing their accessibility level. However, areas located in the central part of Chaoyang District, such as the Dongba Sub-district, showed a decreased accessibility level compared to walking and cycling modes. This could be as the number of parks accessible by car in each community is similar, but the central region exhibits a greater concentration of population, resulting in a slightly lower per capita distribution of park resources compared with other regions.

### Park equity analysis

#### Measurement of park equity

**Figure 6:**
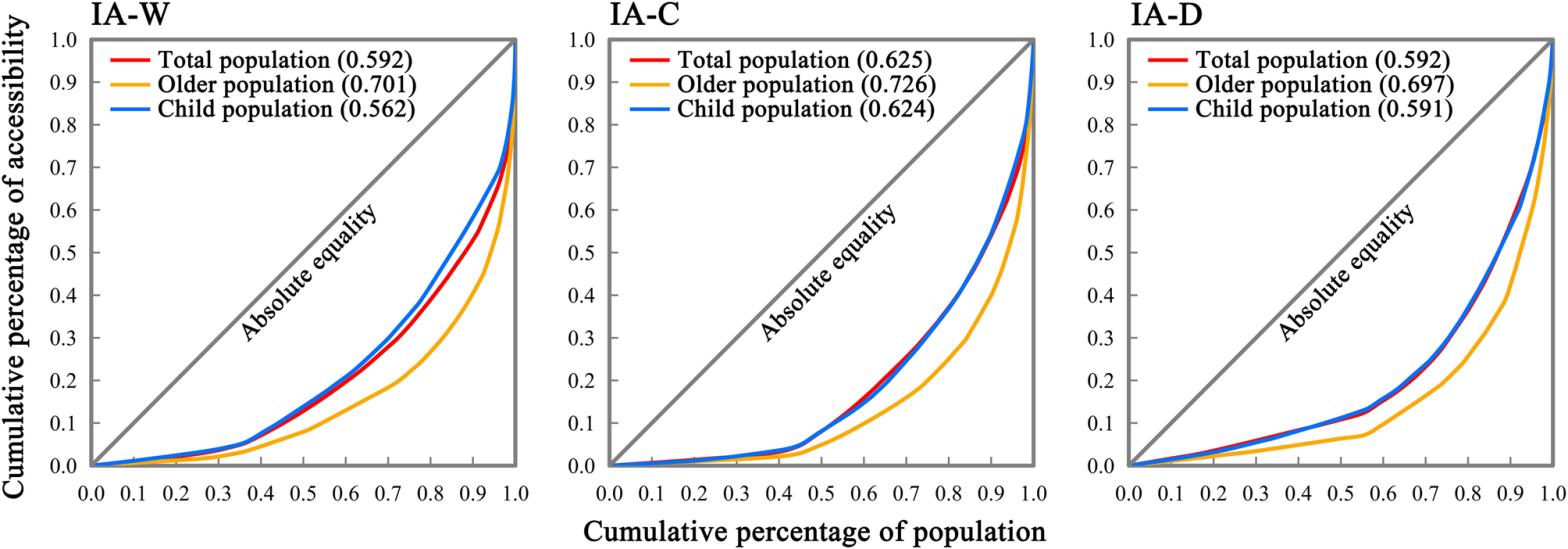
Lorenz curves of park improved accessibility for different population groups.

Drawing on the cumulative percentage of IA under different modes of travel, a Lorenz curve was plotted, and the Gini coefficient was calculated to explore the corresponding distribution of park resources (Fig. 6). The results indicate that the equity of park distribution in the examined region is generally limited, with greater curve distortion in the cycling transport mode specifically. The Gini coefficients for all modes were above 0.600, indicating a notable discrepancy in the allocation of park resources in Chaoyang District. Among the three transportation modes covered, the Lorenz curves for children were closest to the line of absolute equality, with Gini coefficients of 0.561,585,794 for walking, 0.624,461,074 for cycling, and 0.590,679,821 for driving, respectively. The next closest were the curves for the total population, with Gini coefficients of 0.591,972,493 for walking, 0.624,852,273 for cycling, and 0.592,396,953 for driving. The Lorenz curves for older residents showed the greatest distortion, with Gini coefficients of 0.701,143,459 for walking, 0.725,670,406 for cycling, and 0.696,781,465 for driving. This result indicates that the supply–demand match of park resources is more balanced among children, while the distribution of parks is extremely unequal for older residents.

#### Analysis of park supply and demand match

**Figure 7:**
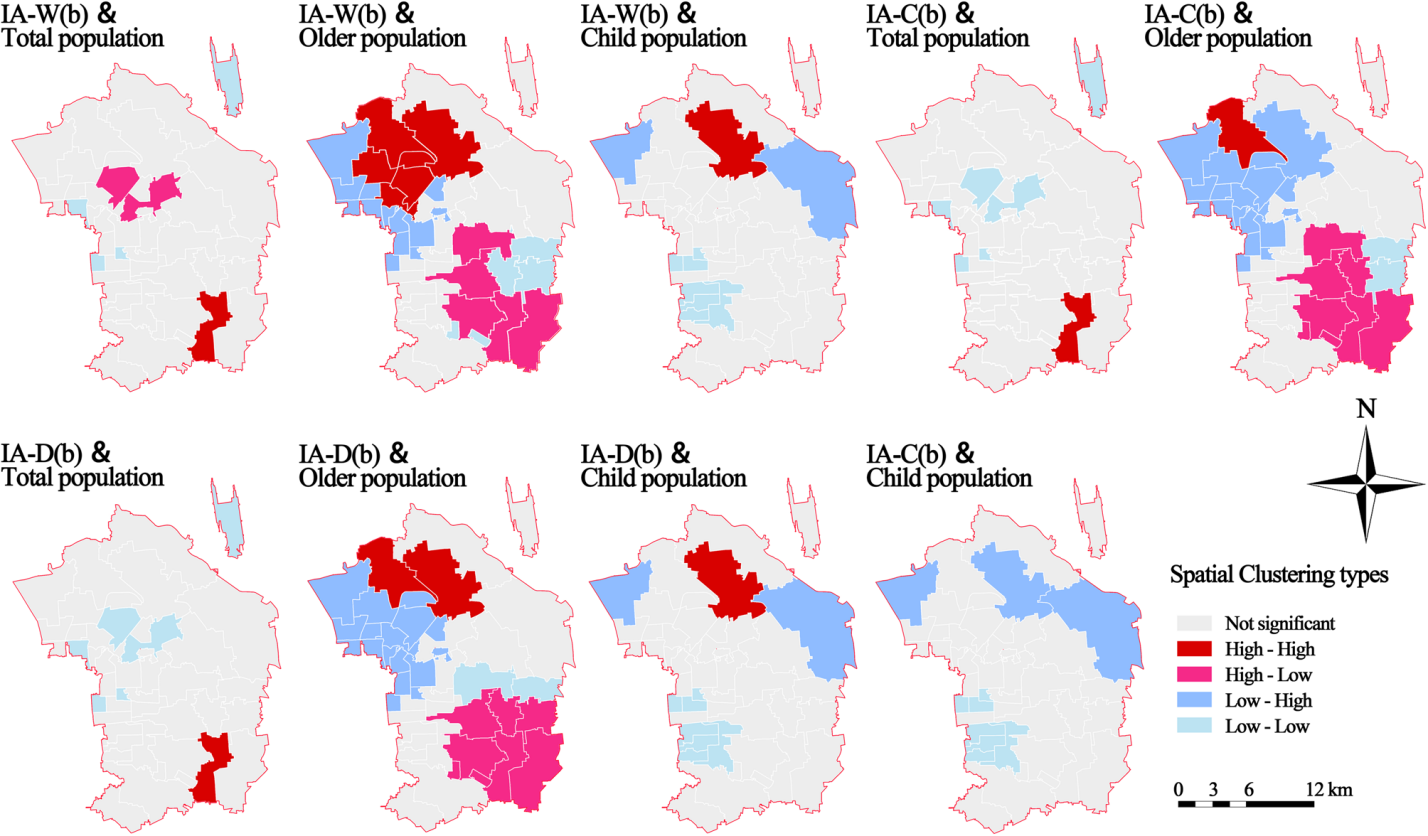
Bivariate local Moran’s I clustering maps.

The results indicate that in certain blocks within Chaoyang District, the correlation between park accessibility and the total population is not particularly noteworthy. Under different modes of travel, the Dougezhuang Subdistrict showed a positive spatial match, indicating that the supply of parks and population demand are relatively high. In contrast, blocks such as Capital Airport Street, Heping Street, Tuanjiehu Street, and Chaowai Street exhibited a negative spatial match, indicating that both supply and demand are limited in terms of quantity. However, with the convenience of different travel modes, blocks such as Wangjing Street and Jiangtai Subdistrict have shifted from positive to negative mismatches (Fig. 7).

There a clear spatial mismatch was found between accessibility and the older population. Blocks with positive mismatches were predominantly found to reside within the southeastern section of Chaoyang District, where the older population is relatively low but park resources are excessive. Blocks with negative mismatches were mainly distributed on the western side of Chaoyang District, near the core area of Beijing, where there is a greater number of older residents and insufficient park resources. Blocks with positive matches were distributed in the northwest, where the supply of parks more closely matches the number of older residents. Blocks with negative matches were located in the eastern part of Chaoyang District, where both parks and older residents are scarce.

Numerous blocks were detected with negative matches between accessibility and child population; these were predominantly located in the southwest near the core area of Beijing. In this area, there is a lower supply of parks and smaller demand from the child population. Blocks such as the Olympic Village and Jinzhan Subdistricts exhibited negative spatial mismatches, indicating a lower supply of parks despite a higher level of demand from the child population. The Cuigezhuang Subdistrict showed a positive match under the walking and driving modes but a negative match under the cycling mode of transportation.

## Discussion

### Exploring factors affecting the recreational quality of urban parks in Chaoyang District

The results indicated that visitors exhibited heightened concern regarding park ticket pricing and recreational activities. Free admission and a diverse range of activities were more likely to provide visitors with positive experiences. Positive evaluations were predominantly related to the parks’ natural environment including the flora and artificial architectural landscape. Abundant greenery and fresh air contributed to a comfortable experience, whereas well-maintained facilities and architecture played a pivotal role in enhancing visitor relaxation. The historical and cultural aspects of parks also attract visitors, as they endow the landscape with a sense of uniqueness and cultural value^62^. Negative evaluations were primarily related to park management issues, encompassing concerns such as discourteous staff attitudes, complicated ticket reservation procedures, high fees, and inadequately maintained public amenities such as restrooms. These problems can create feelings of embarrassment and frustration among visitors, negatively affecting their recreational experience. This study also found that the “pandemic”(疫情) was a key concern for visitors. This may be because the pandemic has significantly affected park operations and management, making parks with free-entry and those that are well-managed more popular among visitors. The recurring mention of the term “Internet celebrity” (网红) implies that the proliferation of social media and online content wields a considerable influence on the allure of parks. However, evaluations of popular parks vary, indicating that a park’s intrinsic recreational quality exerts a more pronounced impact on visitors’ experiences^63^. In addition to overall park quality, the proximity and available modes of transportation also affected visitors’ evaluations, highlighting the importance of park accessibility as a key concern for visitors.

### Advantages of improved accessibility

Previous studies have incorporated park quality into their assessment of urban park accessibility^24^. This study considers recreational quality scores and integrates them into the accessibility calculation using the improved accessibility index to represent the park resources available to residents. The research findings suggest that park sentiment scores exhibit no discernible correlation with popularity and size. However, the number of reviews had a robust positive correlation with park size. This indicates that traditional accessibility calculations relying only on park size can, to a certain extent, represent the allure of a park but are insufficient to represent the supply of park resources. This improved approach quantifies the recreational quality of parks using sentiment scores from textual evaluations of park review networks. The recreational quality index was then utilised to adjust the parameters of park resource supplies. By integrating real evaluations of park recreational quality by visitors, this approach more authentically captures the true supply capacity of park resources, surpassing the limitations associated with sole reliance on park size. This improvement in the calculation method enhances the accuracy of the results.

### Spatial differentiation of park accessibility levels in Chaoyang District

The spatial differentiation of park accessibility levels in Chaoyang District is quite pronounced, and both the Lorenz curves and Gini coefficients for different travel modes indicate an imbalance in resource distribution. The issue of unfairness was particularly pronounced with regard to the cycling mode. The reasons for reduced accessibility in some areas can be summarised as follows: (1) Park resources are inadequate to meet the population demand. Communities near the core area of Beijing have reduced accessibility, possibly owing to their proximity to the area, which is characterised by high-density development. Despite parks being closer to these communities, the excessive population density, coupled with limited park resources, contributes to lower overall accessibility^64^. (2) Difficulty accessing park resources. Capital Airport Street has the lowest level of park accessibility. This is likely because even though the density of residents in this region is relatively small, the distance from parks is greater, and there are fewer transportation routes available, which hinders residents from enjoying park resources^65,66^. (3) Poor park resources. Communities near small parks that lacked sentiment scores had lower accessibility. Insufficient and low-quality greenspace resources fail to meet population demands.

Communities with better park accessibility were primarily located in blocks with high park recreational quality, larger park areas, and a greater number of parks. Observations have revealed that areas with high accessibility are mostly concentrated near Beijing’s first greenbelt^67^. This greenbelt, which forms an urban park ring, has the largest proportion in Chaoyang District. Serving as an ecological barrier for the city, it plays a key role in controlling urban development and provides high ecological service value^68^. The construction of the urban park ring offers residents increased chances to connect with natural surroundings.

### The distribution of urban park resources is inequitable

Urban parks play a significant role as spaces for recreation and amusement, catering to both older citizens and children. However, owing to the respective limitations of both of these population demographics, they are both relatively disadvantaged in this regard. From the perspective of providing adequate care, older residents and children should be key populations of concern when addressing the issue of equity distribution in urban parks^69,70^. Existing research has shown that urban parks not only improve the health of older residents, reducing the incidence and mortality rates of certain diseases, they also provide many broader physiological and psychological benefits^59,71^. Unfortunately, the distribution of park resources among the older residents in Chaoyang District shows the most severe polarisation, with the majority of older people having access to only a few urban parks. Limited content was found in the park evaluation texts analysed in this study relating to the older population, despite many countries facing the challenge of an aging population. Therefore, park planning and design should prioritise placing a greater focus on the older population. Compared to the total population, the distribution of urban park resources among children is more balanced. Children are a significant beneficiary group of parks, as urban parks play a vital role in maintaining children’s physical and mental health and promoting cognitive development^72^. A higher level of attention was paid in the park reviews covered in this study to children and the availability of children's playground facilities, indicating that children's needs should be a key consideration in park planning and design. Similarly, the allocation of urban park resources among the entire population lacks fairness, with a small percentage of people occupying the majority of greenspace resources. Thus, it can be clearly stated that the supply and utilisation rate of park resources in Chaoyang District needs improvement.

### Optimisation suggestions for urban parks in Chaoyang District

Enhancing the quality of urban park recreation. One of the important reasons for this inequitable distribution is the difference in park resources. To some extent, improving park quality can alleviate the problem of insufficient park supply. Based on this analysis of park reviews, measures such as optimising green space quality, improving park management models, enriching activities within parks, and maintaining public service facilities can enhance park recreational quality. Additionally, it is important to consider the greenspace needs of vulnerable groups, such as older residents and children, by providing accessible facilities and creating older adult and child-friendly parks.

Optimizing the horizontal spatial arrangement of urban parks is crucial. There exists a significant disparity in the horizontal spatial accessibility of urban parks in Chaoyang District, which has created an uneven distribution of park resources. Rational planning of the horizontal spatial layout of urban parks can help mitigate inequities in resource distribution. However, Chaoyang District is a densely developed urban area with limited land resources, making it difficult to expand urban parks extensively. One potential approach is to conduct research and identify unused or abandoned areas to create pocket parks and micro-green spaces that can provide additional green resources for residents and improve the accessibility and equitable distribution of urban parks. This can also serve to enhance the connectivity of green spaces, significantly optimising the urban ecological environment.

Industrial development in residential areas. In Chaoyang District, an imbalance between the supply and demand of urban park resources and the population is mainly observed in the blocks located near the core area of Beijing and near the outer ring road. This disparity could be attributed to the large population near the core area of Beijing, which has a scarcity of parks. By comparison, the south-eastern part of Chaoyang has a smaller population, but a higher concentration of parks with better recreational quality. Engaging in industrial development in the residential areas on the outskirts of Beijing can effectively increase the utilisation rate of parks, more widely disperse the population near the core area, and alleviate the supply pressure on urban parks within the inner ring (Supplementary Information).

## Conclusions

Urban park accessibility is a crucial aspect of environmental equity. This study, using Chaoyang District in Beijing as a case study, incorporates web-based sentiment scores and travel patterns into the traditional G2SFCA accessibility calculation for urban parks. This method provides a more comprehensive and accurate reflection of urban park supply capacity while examining the impact of park recreational quality and travel patterns on accessibility. Furthermore, by analysing park review texts, valuable insights from both residents and visitors were obtained, enabling a holistic understanding of the key factors influencing park recreational quality. This, in turn, enables more tailored recommendations for optimising future park spatial layouts and quality improvements. The study yields several key findings: (1) users prioritise park management and expect high standards of convenience and cleanliness of public facilities; (2) recreational quality impacts park distribution equity, with improved accessibility near Beijing’s first greenbelt; (3) park resources in Chaoyang District are distributed unequally among various social groups, with significant spatial disparities in the northwest and southeast; (4) enhancing park layout equity can be achieved through repurposing idle spaces, establishing pocket parks, and other small-scale green areas, and improving recreational quality; and (5) urban park planning and construction should consider the specific needs of vulnerable groups such as older residents and children. In conclusion, by incorporating the improved accessibility analysis method based on tourist reviews, this study considers the impact of park recreational quality on park attractiveness, enabling a more accurate and objective analysis of park accessibility. This approach demonstrates a certain level of innovation and universality in its application. However, the analysis method employed in this study faces several challenges in real-time applications. This paper relied solely on sentiment scores from online media as a quantifiable indicator of recreational quality, posing limitations. Additionally, the simulation of different travel patterns only considered time costs and did not explore the influence of different types of roads on travel. The SnowNLP model requires a large amount of training text, and sentiment analysis is greatly influenced by the selection and judgment of training text. Further discussions are needed to determine which computer models can more accurately and reasonably analyze the emotional states of tourist reviews, how to collect more authentic online evaluation data, and how to ensure the real-time nature of the geographical data required for accessibility analysis.

### Supplementary Information

Supplementary Information.

## Data Availability

The data presented in this study are available on request from the corresponding author.
