# Supplementary material for: Assessing urban park equity in Chaoyang District, Beijing using online review data
Source: Sci Rep. 2024 Jan 12;14:1160. doi: 10.1038/s41598-024-51239-9 (PMC10786826; doi:10.1038/s41598-024-51239-9)
Supplement: Supplementary file 1 — Supplementary Information. [file 41598_2024_51239_MOESM1_ESM.docx]

Figure data source and processing software

| **Tape** | **Name** | **Sources** | **Description** |
| --- | --- | --- | --- |
| Basic datas | Traffic networks | Bigemap GIS Office （http://www.bigemap.com/） | Vector data; 2022 |
|  | Administrative boundaries |  |  |
|  | POI of Communities |  |  |
|  | Park ranges | Beijing Municipal Forestry and Parks Bureau (Office of Beijing Greening Commission) (https://yllhj.beijing.gov.cn/) |  |
| Processing softwares | ArcGIS | https://www.arcgis.com/ | Geographic information processing software |
|  | Photoshop | https://www.adobe.com/in/products/photoshop.html | Drawing software |
